# Acute Lung Injury after Cardiopulmonary Resuscitation: A Narrative Review

**DOI:** 10.3390/jcm13092498

**Published:** 2024-04-24

**Authors:** Giuseppe Marchese, Elisabetta Bungaro, Aurora Magliocca, Francesca Fumagalli, Giulia Merigo, Federico Semeraro, Elisa Mereto, Giovanni Babini, Erik Roman-Pognuz, Giuseppe Stirparo, Alberto Cucino, Giuseppe Ristagno

**Affiliations:** 1 UOC Anestesia e Rianimazione, Ospedale Nuovo di Legnano, ASST Ovest Milanese, 20025 Legnano, Italy; 2 Department of Pathophysiology and Transplantation, University of Milan, 20122 Milan, Italyaurora.magliocca@unimi.it (A.M.); elisa.mereto@gmail.com (E.M.); 3 Department of Anesthesiology, Intensive Care and Emergency, Fondazione IRCCS Ca’ Granda Ospedale Maggiore Policlinico, 20122 Milan, Italy; giulia.merigo@unimi.it (G.M.);; 4 Department of Acute Brain and Cardiovascular Injury, Istituto di Ricerche Farmacologiche Mario Negri IRCCS, 20122 Milan, Italy; 5 Department of Biomedical Sciences for Health, University of Milan, 20122 Milan, Italy; 6 Department of Anesthesia, Intensive Care and Prehospital Emergency, Maggiore Hospital Carlo Alberto Pizzardi, 40133 Bologna, Italy; 7 Department of Anesthesia and Intensive Care, University of Trieste, 34127 Trieste, Italy; 8 Agenzia Regionale Emergenza Urgenza—AREU, 20122 Milan, Italy; 9 Department of Anaesthesia and Intensive Care Medicine, APSS, Provincia Autonoma di Trento, 38121 Trento, Italy; alberto.cucino@gmail.com

**Keywords:** cardiac arrest, cardiopulmonary resuscitation, lung injury, lung edema

## Abstract

Although cardiopulmonary resuscitation (CPR) includes lifesaving maneuvers, it might be associated with a wide spectrum of iatrogenic injuries. Among these, acute lung injury (ALI) is frequent and yields significant challenges to post-cardiac arrest recovery. Understanding the relationship between CPR and ALI is determinant for refining resuscitation techniques and improving patient outcomes. This review aims to analyze the existing literature on ALI following CPR, emphasizing prevalence, clinical implications, and contributing factors. The review seeks to elucidate the pathogenesis of ALI in the context of CPR, assess the efficacy of CPR techniques and ventilation strategies, and explore their impact on post-cardiac arrest outcomes. CPR-related injuries, ranging from skeletal fractures to severe internal organ damage, underscore the complexity of managing post-cardiac arrest patients. Chest compression, particularly when prolonged and vigorous, i.e., mechanical compression, appears to be a crucial factor contributing to ALI, with the concept of cardiopulmonary resuscitation-associated lung edema (CRALE) gaining prominence. Ventilation strategies during CPR and post-cardiac arrest syndrome also play pivotal roles in ALI development. The recognition of CPR-related lung injuries, especially CRALE and ALI, highlights the need for research on optimizing CPR techniques and tailoring ventilation strategies during and after resuscitation.

## 1. Introduction

Cardiac arrest stands as a formidable challenge in global health, currently ranking as the third leading cause of death worldwide [1]. The recent EuReCa TWO study reported an incidence of out-of-hospital cardiac arrest (OHCA) in Europe of 89 events per 100,000 inhabitants per year. Despite the high frequency of OHCA, outcomes often remain bleak, with only about one-third of patients achieving the return of spontaneous circulation (ROSC), and the survival rate dwindling further, with only 8% of individuals being discharged alive from hospital [2].

Cardiopulmonary resuscitation (CPR) is a life-saving emergency procedure aiming to restore oxygenated blood flow to the brain and heart during cardiac arrest. However, this essential intervention is not without significant drawbacks. Indeed, resuscitation procedures frequently generate a wide spectrum of iatrogenic injuries to the resuscitated persons [3]. These injuries are prevalent, with almost every patient undergoing CPR experiencing some form of damage, ranging from relatively minor issues, such as simple bruises or skin abrasions, to more severe and concerning complications like thoracic skeletal fractures and organ injuries [4]. While the former are more common and tend to have a limited clinical impact, the latter, particularly severe organ injuries, can pose life-threatening conditions and might significantly affect patient outcomes [5,6,7,8,9,10,11,12].

One of the most significant complications potentially encountered after CPR is acute lung injury (ALI), which can lead to respiratory failure and eventually progress to acute respiratory distress syndrome (ARDS) [13]. Post-CPR ALI is a significant yet under-discussed complication in cardiac arrest recovery, traditionally associated with various risk factors. These include post-cardiac arrest syndrome (PCAS), which encompasses a range of pathological processes triggered by the ischemia–reperfusion injury inherent in cardiac arrest and resuscitation [14]. Other contributing factors to ALI include pulmonary ischemia–reperfusion or aspiration pneumonia, often occurring during or after the cardiac arrest; direct lung contusions resulting from the physical impact of CPR; post-resuscitation inflammation; pulmonary infections that can develop during hospitalization; and ventilator-induced lung injury (VILI), a consequence of mechanical ventilation frequently encountered in intensive care unit (ICU) patients [15,16,17].

The recent literature has begun to cast a spotlight on CPR itself as a pivotal moment in the development of ALI. The act of performing chest compression, a fundamental component of CPR, is thought to be a primary contributor to ALI [17]. This contribution is not due merely to the direct traumatic injury inflicted upon the lungs and the chest wall, which has long been recognized, but also to the acute lung edema that might be provoked by swings in intrathoracic pressure during the resuscitation process. These insights have been increasingly supported by emerging studies, including those focusing on cardiopulmonary resuscitation-associated lung edema (CRALE) [17]. Such studies have begun to unravel the complex interplay between the mechanical aspects of CPR and the subsequent physiological responses, particularly concerning lung health [17,18].

The ventilation strategy adopted during CPR and in the post-ROSC period might also concur in the development of ALI. While current guidelines universally recommend the provision of ventilation during CPR, there remains a lack of consensus or clear guidance on the optimal ventilation strategy [19]. This ambiguity extends into the realm of post-resuscitation care, where recommendations are scarce and often non-specific [14]. Given the significant impact of ALI on post-cardiac arrest outcomes and the potential role of CPR in its development, there is a pressing need for a deeper understanding in this area [14,18,19,20].

This review aims to critically analyze and synthesize the existing literature regarding ALI following CPR, shedding light on its prevalence, while emphasizing its clinical implications. Understanding this correlation is determinant in enhancing post-cardiac arrest care and outcomes. Thus, the review seeks to illuminate the current understanding of ALI pathogenesis in the context of CPR, evaluate the effectiveness of various CPR techniques and ventilation strategies, and explore their implications for improving patient outcomes after cardiac arrest.

## 2. CPR-Related Injuries

While CPR is a lifesaving maneuver, its history, spanning more than six decades, is also marked by its potential to cause injuries [21]. Indeed, the practice of chest compression, a cornerstone of CPR, has long been associated with a variety of injuries, a concern that dates back to the early days of closed-chest cardiac massage [22]. A multitude of studies have been dedicated to quantifying CPR-related injuries and identifying potential risk factors. The variety and incidence of the different CPR-related injuries show considerable variability and are summarized in Table 1. The discrepancy among the studies could partly be attributed to the underdiagnosis of these injuries. Traditional diagnostic tools like chest X-ray may not detect all cases of thoracic skeletal fractures or lung contusions, whereas more sensitive methods like computed tomography (CT) scan or autopsy, when performed, reveal a clearer picture [23,24]. Rib and sternal fractures are the most reported injuries following CPR. However, their impact on the outcomes of post-cardiac arrest patients is relatively limited compared to more severe internal organ injuries [5,6,7,8]. Indeed, intrathoracic injuries, though less frequent, are of greater concern because they can be life-threatening and may significantly impede the effectiveness of resuscitative efforts [4,9,10,11,12].

The risk factors for developing CPR-related injuries are variable. They include patient-specific factors like age, sex, and chest wall dimensions, as well as CPR-specific variables like the duration of the CPR, the depth of compression, and whether manual (CC) or mechanical chest compressions (mCC) are performed [4,5,10,11,17,23]. The evolution of CPR guidelines might have also impacted on the injury rates. In 2010, guidelines increased the recommended depth of chest compression from 4–5 cm to 5–6 cm, while introducing a faster rate of 100–120 compressions per minute. Although these changes aimed at improving hemodynamic support and ultimately patient outcomes, they have also been associated with a higher incidence of thoracic injuries [25,26].

Notably, the introduction of devices generating not only chest compression but also active decompression, i.e., active chest recoil (AD-mCC), raises additional concerns about potentially higher injury rates, although a Cochrane analysis has found no significant difference in serious injuries compared to standard CPR, highlighting, however, the confounding factor of manual chest compression performed in all cases before the deployment of the mechanical device [6,8,23,27]. A recent analysis comparing autopsy-documented injury patterns caused by standard mechanical chest compression vs. mechanical compression with active decompression in 221 OHCAs reported similar skeletal and non-skeletal fractures/injuries in both groups, with, however, a trend towards more injuries with the use of active decompression [28].

The focus of the literature on CPR-related injuries has traditionally been only on skeletal damage. The first study describing the radiological features associated with lung injuries was published in 2013 [24]. This study, involving 44 non-traumatic cardiac arrests, used CT scans, and revealed injuries in a significant number of cases, i.e., in 79.5% of patients. Compared to chest X-rays, CT scans proved to be more sensitive in detecting and characterizing lung injuries. Ground-glass opacities and consolidations were the most common injuries, present in more than 74% of instances. In more than half of the cases, these findings were bilateral, and 83% of the time, the injuries were present in the dependent lung areas [24]. The same results were reported more recently by Yang et al., who performed CT scans within 24 h of ROSC in a cohort of 43 non-traumatic cardiac arrests, demonstrating that bilateral lung contusions were present in all the patients. The radiological findings were consolidation and ground-glass opacity in 93% of the patients, and in almost all the cases, i.e., 95%, lung contusions were present in the dependent regions [29]. Ground-glass opacities further confirmed by histopathological findings were then reported and quantified by CT scan analysis in porcine models of CPR [30]. The variety and incidence of CPR-related lung injuries revealed by chest CT scan are summarized in Table 2. Areas of lung consolidation with serofibrinous exudate, hemorrhage, and inflammatory cells were also described. Moreover, alveolar collapse, alveolar distension, intra-alveolar and peri-bronchiolar hemorrhage were frequently observed [16,30,31].

**Table 1:** Reported incidence of cardiopulmonary resuscitation-related thorax, lung, and organ injuries.

Reference	Study Design	Rib Fractures	Sternal Fractures	Lung Contusions	Pneumo-Thorax	Effusion or Hemothorax	Hemo-Pericardium	Liver Lesions	Spleen Lesions
Ihnát Rudinská 2016 [4]	Prospective analysis of 80 autopsies after OHCA	74%	66%	31%	-	0.5%	9%	-	-
Hoke 2004 [5]	Review study including 16 studies: 14 studies with prospective and/or retrospective analysis of 2036 autopsies after IHCA and OHCA One study with prospective analysis of autopsies and/or chest radiographs and/or clinical data from 145 IHCA cases One study with analysis of 173 chest radiographs after OHCA	13–97%	1–43%		1.3–3%	0.8–8.7%	1.1–8.4%	0.8–4.3%	0.3–2.6%
Smekal 2014 [6]	Prospective multicenter study of 222 autopsies after OHCA	CC 64.6% mCC 78.8%	CC 54.2% mCC 58.3%	-	-	-	-	-	-
Karasek 2022 [7]	Retrospective analysis of 628 autopsies after IHCA and OHCA	94.6%	62.4%	9.9%	-	-	-	2.5%	1.8%
Ondruschka 2018 [8]	Retrospective analysis of 614 autopsies after IHCA and OHCA	CC 59.7% mCC 74.3%	CC 27.2% mCC 47.8%	CC 0.04% mCC 18.6%	CC 0.6% mCC 6.2%	CC 1.2% mCC 8.9%	CC 0.6% mCC 2.7%	CC 1.4% mCC 9.7%	-
Miller 2014 [10]	Systematic review with pooled data analysis from 27 studies. Injuries detected by chest radiographs and/or CT scan and/or ultrasound after IHCA and OHCA	31.2% CC 25.9% mCC 32.7%	15.1% CC 8.5% mCC 25.8%	1.7% CC 0% mCC 2.8%	2.5% CC 2.1% mCC 2.6%	2.1% CC 3.9% mCC 6.3%	7.5%	-	-
Ram 2018 [23]	Review including 23 studies with autopsies and/or chest radiographs and/or CT scans from > 53.000 non-traumatic and traumatic IHCA and OHCA	27–90%	4–21%	1.7–41%	-	-	7.5%	0.6–3%	-
Lafuente-Lafuente 2013 [27]	Analysis of autopsy-documented injury caused by standard mCC vs. AD-mCC after 221 OHCA	69% mCC 67% AD-mCC 72%	46% mCC 44% AD-mCC 48%	5% mCC 3% AD-mCC 8%		18% mCC16% AD-mCC 20%		2% mCC 0% AD-mCC 4%	1% mCC 2% AD-mCC 0%

CC, manual chest compression; CT, computed tomography; mCC, mechanical chest compression; AD-mCC, mechanical chest compression with active decompression; OHCA, out-of-hospital cardiac arrest; IHCA, in-hospital cardiac arrest.

**Table 2:** CPR-induced lung injuries revealed by chest computed tomography scan [24,29,30].

Lung Injury	Incidence
Consolidations only	0–14%
Ground-glass opacities only	0–11%
Concurrent consolidations and ground-glass opacities	74–100%
Unilateral (only right or left lung) injuries	0–46%
Bilateral (both right and left lung) injuries	54–100%
Location in the dependent lung regions	83–95%

Serious thoracic and lung injuries are also thought to adversely affect hemodynamics during CPR itself. They can reduce intrathoracic negative pressure and functional residual capacity during the decompression phase of chest compression, thereby diminishing venous return to the heart, ultimately impairing the cardiac output generated by chest compression. This detrimental hemodynamic effect can be further increased by chest leaning and/or hyperventilation during CPR [12].

Evidence of the central role of lung injury in resuscitated patients is further supported by observations that the respiratory system is often the most compromised organ apparatus following cardiac arrest, together with the cardiovascular one [14,18,32]. The deterioration of these systems, as assessed by the Sequential Organ Failure Assessment (SOFA) score, has been identified as an independent predictor of post-cardiac arrest mortality [32]. A retrospective cohort study enrolling 600 OHCA survivors who required mechanical ventilation, revealed that nearly half of these patients met the Berlin criteria for ARDS within 48 h of hospital admission [33]. In these patients, the development of ARDS was linked to higher hospital mortality, longer ICU stays, more days on ventilation, and a lower likelihood of recovering with full neurological function [13].

In the context of traumatic cardiac arrest patients, the literature suggests that ALI develops in a significant portion of patients with isolated pulmonary contusions, and the risk escalates dramatically in patients with additional injuries. Pneumonia develops in 20% of these patients [34]. The pathological mechanisms involve an increase in secretions in the traumatized area, reduced clearance due to ciliary inactivation and bronchial edema, and blood filling in the alveolar spaces, thus providing a medium for bacterial growth. The activation of inflammatory pathways further exacerbates tissue edema, hampers surfactant function, and promotes atelectasis [34,35].

In summary, while CPR is a critical intervention in the management of cardiac arrest, it is not without risks. Understanding the spectrum of injuries associated with CPR, particularly those affecting the thorax and lungs, is essential for improving resuscitation techniques and post-resuscitation care. This understanding also underscores the need for continued research and the refinement of CPR guidelines to balance the benefits of resuscitation with the minimization of injury risk.

**Figure 1:**
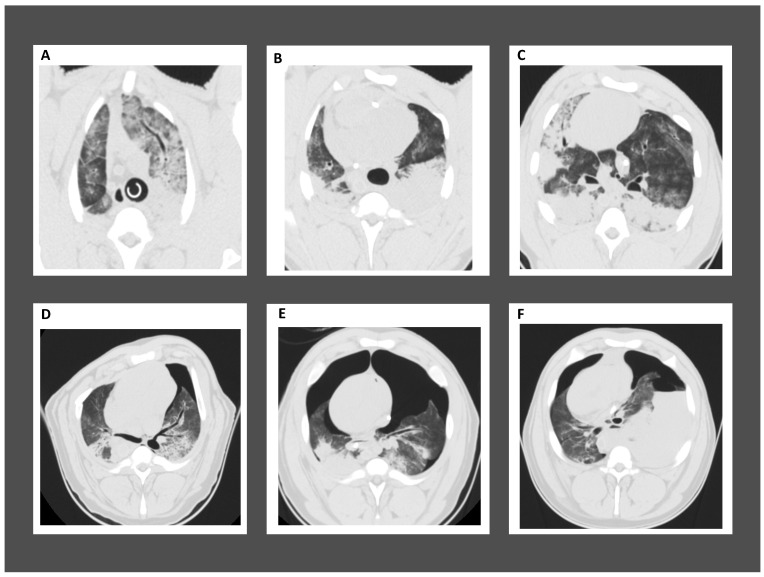
Representative computed tomographic images of lung injuries following cardiopulmonary resuscitation: (**A**,**B**) cranial and (**C**) caudal lung lobes of pigs with cardiopulmonary resuscitation-associated lung edema (CRALE); (**D**) right pneumothorax with left rib fracture; (**E**) bilateral pneumothorax; (**F**) bilateral pneumothorax with right hemothorax.

Figure 1 reports CT scans with different patterns of thorax and lung injuries obtained from experimental studies performed in the authors’ laboratory in a porcine model of cardiac arrest with 20 min of continuous mechanical CC (LUCAS^®^ Stryker, Lund, Sweden) and asynchronous mechanical ventilation [17].

## 3. ALI in Post-Cardiac Arrest Patients

ALI is a significant complication in patients who have suffered a cardiac arrest, with many mechanisms accounting for its development beyond the direct injury caused by CPR, including, firstly, PCAS [15,16,17,18,19,36]. PCAS is a complex condition that arises following ROSC and is characterized, among many aspects, by an intense ischemia–reperfusion injury, which shares numerous pathophysiological similarities with sepsis, including oxidative stress, coagulopathy, and widespread inflammation, culminating in multiorgan dysfunction [14,37]. In the lungs, both PCAS and sepsis can lead to the altered permeability of alveolar endothelial and epithelial barriers, a key pathway to the development of ARDS. The management of pulmonary dysfunction in these conditions is thus a critical aspect of post-resuscitation care [37,38].

Aspiration pneumonia is another crucial risk factor. Nearly 30% of cardiac arrest patients experience emesis, leading to an increased risk of aspiration [39]. This risk is compounded using temperature control at hypothermic level, which, while potentially beneficial, has been associated with an elevated risk of ventilator-associated pneumonia [40,41]. Consequently, the incidence of early-onset pneumonia is notably high among post-cardiac arrest patients, underscoring the need for the vigilant monitoring and management of pulmonary complications in this vulnerable group [42,43].

Cardiac failure in the immediate post-ROSC period is another common phenomenon [14,37,44]. A significant proportion of patients exhibit marked left ventricular systolic dysfunction, and studies have documented hemodynamic instability, particularly in the first 24 h following cardiac arrest. These patients often require substantial volume expansion to maintain hemodynamic stability, placing them at high risk of developing and/or worsening cardiogenic lung edema. Indeed, a transient decrease in the cardiac index, i.e., 2.05 L/min per m^2^, requiring a median of 8 L of volume expansion over the first 72 h post-ROSC, has been reported. Thus, cardiac dysfunction and its management in the acute post-resuscitation phase are other crucial determinants of lung health and the risk of ALI [14,44,45,46].

Finally, while tracheal intubation and mechanical ventilation are cornerstones of managing comatose cardiac arrest survivors, they are not without risks [14]. Mechanical ventilation, specifically, might lead to the occurrence of VILI, further complicating the pulmonary picture in these already-vulnerable patients [47].

## 4. Cardiopulmonary Resuscitation-Associated Lung Edema (CRALE)

The recent literature has increasingly focused on CPR, particularly on chest compression, as a critical factor in the development of ALI. While historically, post-CPR lung injury was attributed to the direct trauma inflicted by compression on the lungs and chest wall, emerging animal and clinical observations have suggested that the rapid changes in intrathoracic pressure occurring during chest compression and decompression might lead to the generation of acute lung edema, a condition recently termed CRALE [17].

In a pivotal study conducted in 2020, Magliocca et al. explored the concept of CRALE through a translational approach involving both a porcine model and a cohort of OHCA patients undergoing mechanical or manual chest compression during CPR. Lung CT scans after resuscitation were analyzed together with changes in respiratory mechanics and gas exchanges, comparing results between mechanical and manual compression. The investigation revealed, in both animals and humans for the first time, that upon mechanical CPR, a higher lung weight, lower arterial oxygenation, reduced respiratory system compliance, and a higher incidence of abnormal lung density were present compared to manual compression. The severity of CRALE was related to the duration of the CPR and, particularly, mechanical CPR exacerbated this lung injury after cardiac arrest. Further mechanistic analyses demonstrated a correlation between chest compression-induced variations in the right atrial pressure and the severity of CRALE, suggesting that negative intrathoracic pressure swings during CPR are instrumental in causing the appearance and the severity of this event. This mechanism was further substantiated by the observation of increased hydrostatic pressure gradients leading to transcapillary flow with alveolar flooding and inflammation, confirmed at histopathology [17]. Moreover, earlier studies have described a rapid increase in pulmonary artery diastolic pressure during CPR, which rapidly decreased after the cessation of resuscitation, being another possible event contributing to the development of post-resuscitation pulmonary edema [48].

**Figure 2:**
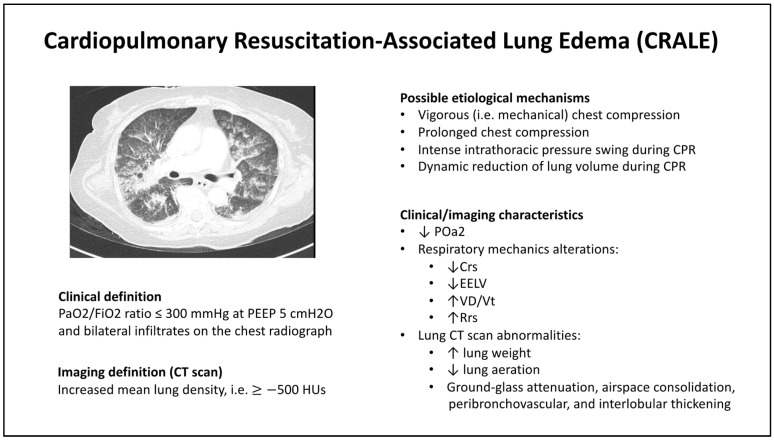
Cardiopulmonary resuscitation-associated lung edema. Crs, respiratory system compliance; EELV, end-expiratory lung volume; HU, Hounsfield unit; PaO2/FiO2, oxygen arterial partial pressure/oxygen inspiratory fraction; Rrs, respiratory system resistance; VD/Vt, dead space fraction.

Building upon these findings, a subsequent clinical study clearly defined the conditions of CRALE as the presence of a PaO2/FiO2 ratio ≤ 300 mmHg at positive end-expiratory pressure (PEEP) 5 cmH2O and bilateral infiltrates on the chest radiograph, and extended observations on the respiratory characteristics of cardiac arrest patients [18]. By employing esophageal catheters, the partitioned respiratory mechanics and dead space fraction (VD/Vt) were studied and differentiated in patients with and without CRALE. Patients suffering from CRALE exhibited lower respiratory system compliance and end-expiratory lung volume (EELV), coupled with a higher VD/Vt and respiratory system resistance. Notably, the chest wall compliance did not differ significantly between the two groups, indicating that the primary cause of deteriorating respiratory mechanics in CRALE patients was lung-related. These insights provide a nuanced understanding of the complex interplay between CPR, particularly chest compression, and lung health. These findings underscore the need for careful consideration of CPR techniques and the potential need for tailored ventilation strategies in the immediate post-resuscitation period to mitigate the risk of CRALE and subsequent ALI and to improve patient outcomes [14,17,18,49,50]. The definitions and characteristics of CRALE are detailed in Figure 2.

In conclusion, the understanding of ALI following cardiac arrest has evolved significantly. While traditional risk factors like PCAS, aspiration pneumonia, and VILI remain critical, the role of CPR, and especially the mechanical aspects of chest compression, have emerged as a key area of focus. The implications of these findings are profound, highlighting the need for ongoing research, the refinement of CPR protocols, and targeted post-resuscitation care strategies to optimize patient recovery and minimize the risk of severe pulmonary complications.

## 5. Ventilation Strategies and ALI

The ventilation strategy during cardiac arrest is a critical aspect of CPR; however, due to the low level of scientific evidence, no consensus on the best approach remains. Current guidelines recommend maintaining a high fraction of inspired oxygen (FiO2) and ventilating at a rate of 10 breaths per minute, without specific recommendations on ventilation modality, i.e., a 30:2 compression/ventilation ratio or asynchronous ventilation during continuous chest compressions, mechanical or manual mode, and the use of PEEP, generally thought to increase intrathoracic pressure during CPR and transthoracic impedance during defibrillation [19,20]. Indeed, an international survey targeting physicians frequently involved in CPR maneuvers revealed significant heterogeneity in how ventilation is delivered during CPR across 54 European countries, often diverging significantly from established guidelines [51].

The importance of ventilation during CPR stems from the inadequacy of chest compression alone to generate sufficient passive tidal volumes. Studies have shown that even with adherence to the recommendations for high-quality CPR, the passive tidal volume generated by compression is often minimal, i.e., only 7.5 mL. While the highest recorded compression-generated volume during compression is 45.8 mL, 81% of the measured tidal volumes account for less than 20 mL [52]. Studies in human “Thiel” cadavers highlighted the reduction in lung volume during cardiac arrest due to the loss of rib cage muscle tone, a condition worsened by the mechanical effect of chest compression [53]. Therefore, during ongoing CPR, EELV falls below the closing capacity, finally leading to an airway collapse. Thus, under this condition, even if chest decompression produces a negative intrathoracic pressure, it is not able to generate an inspiratory flow. Hence, lung injury, atelectasis, and congestion could derive from volume reduction along with the mechanical forces employed externally by chest compression. The analysis of the capnogram waveform has suggested that applying a small amount of PEEP (5–10 cmH2O) may prevent this airway closure, increases airway opening index, and enhances the amount of efficient alveolar ventilation produced directly by chest compression alone [53].

Ventilation is essential not only to provide oxygenation, but also for CO_2_ removal from the blood, and thus insufficient ventilation can lead over time to a decrease in serum pH. The resulting acidosis can adversely affect patient outcomes by reducing oxygen–hemoglobin affinity, decreasing cardiac contractility, altering systemic and pulmonary vascular resistances, increasing the risk of cardiac arrhythmias, and reducing the success rate of defibrillation [54,55]. Conversely, positive pressure ventilation, when performed at a high rate or with prolonged intervals, can decrease cardiac preload and output, increase pulmonary vascular resistance, impede right ventricular function, and inversely affect coronary perfusion pressure [56]. Besides increases in intrathoracic pressure, hyperventilation poses also other significant risks, leading to hypocapnia, cerebral vasoconstriction, reduced cerebral perfusion, and, subsequently, poor neurological outcomes [56,57]. Even if the potential harm of hyperventilation during CPR is well known, almost 40% of physicians have confirmed using a ventilation rate greater than 10 breaths/min when using a bag-mask approach [51]. Moreover, in intubated adult OHCA patients, ventilation rates of up to 30 breaths/min have been recorded during CPR performed by trained professional rescuers, leading to a measured positive pressure in the lungs for approximately half of the resuscitation time, with ultimately no survival [56].

Asynchronous ventilation is often delivered after an advanced airway device has been placed, either with a self-inflating balloon or with a mechanical ventilator [50]. However, asynchronous ventilation, when combined with chest compression, could result in increases in peak inspiratory pressure due to the constant and rapid changes in chest wall compliance, raising the risk of lung injury besides challenging the delivery of the required tidal volume [58]. The combination of asynchronous ventilation with the use of mechanical chest compression can further exacerbate this challenge. More than 70% of the interviewed physicians have confirmed experiencing major problems concerning ventilation when delivered through a ventilator during CPR, with the most frequent reported problems including airway pressure alarms (almost 90% of cases), delivery of low tidal volumes, and hemoptysis. Consequently, 32% of the physicians have stated that they always or frequently modify the ventilation practice when using a mechanical compressor and almost 75% revert to self-inflating balloon ventilation when a mechanical ventilator is used [51]. When mechanical ventilation is adopted, there is also an increased risk of alveolar structure overdistention, namely, barotrauma, due to the high inspiratory pressures that could be achieved with simultaneous CC and inspiration. This elevated peak inspiratory pressure may also trigger the ventilator’s maximum pressure limit, resulting in halted tidal volumes and ultimately worse patient oxygenation [58]. In addition, another frequent problem with mechanical ventilation during CPR is the auto-triggering or the inappropriate activation of ventilator delivery due to the setting of the ventilator’s inspiratory pressure- or flow-trigger. Thus, to avoid hyperventilation and the consequent deterioration of gas exchange and hemodynamics, the ventilator inspiratory trigger should be turned off during CPR [58].

The current evidence does not show the superiority of continuous CC with asynchronous ventilation vs. interrupted CC with synchronous ventilation in cardiac arrest outcomes, with an odds ratio (OR) of 1.07 ([95% CI] 0.86–1.32) for ROSC, 1.04 (0.77–1.42) for survival to hospital discharge, and 0.92 (0.84–1.01) for good neurological recovery [59]. Indeed, a large randomized trial enrolling 23.711 OHCA patients led to a ROSC rate of 9.0 vs. 9.7% and survival with good neurological outcomes of 7 vs. 7.7% with continuous compressions with unsynchronized ventilation when compared to a 30:2 CC/ventilation approach, respectively [60].

In addition, the literature does not support any significant benefit of mechanical ventilation over manual ventilation. A recent randomized trial in 60 non-traumatic adult cardiac arrest patients admitted to the emergency department showed no difference in PaO2 (29.0 vs. 36.5 mmHg), ROSC (50 vs. 43%), and survival (37 vs. 30%) between ventilation with a mechanical ventilator (breath rate 10/min, tidal volume 6–7 mL/kg, PEEP 0 cmH2O) or with a bag valve, respectively [61]. Similarly, another recent study confirmed no effect on ROSC (60.3 vs. 50.7%) or survival with good neurological outcomes (15.6 vs. 11.3%) in 150 OHCAs randomized to mechanical ventilation (breath rate 10–12/min, tidal volume 7 mL/kg, PEEP 5 cmH2O) or bag ventilation (10–12 breath/min, O_2_ 15 L/min) [62].

Finally, new mechanical ventilation modes capable of overcoming the above-mentioned limitations related to the use of a ventilator during CPR are currently under evaluation. Particularly, a specific non-synchronized bi-level pressure ventilation mode, called cardiopulmonary ventilation (CPV), has been retrospectively evaluated in comparison to manual bag ventilation in 2566 OHCAs [63]. CPV was associated with an increased probability of ROSC (49 vs. 42.8%; OR 2.16 (1.37–3.41)), but not with improved neurological outcomes (10.7 vs. 9.5%; OR: 1.44 (0.72–2.89)). Thus, further randomized trials are now needed to prove the effectiveness of this new mechanical ventilation strategy for CPR.

Guidelines on post-resuscitation care suggest adopting a protective ventilation strategy to prevent VILI and aiming for a tidal volume of 4–8 mL/kg/ideal body weight to avoid overdistention; a PEEP level guided by the monitoring of airway pressures as a surrogate of compliance so that excessive pressure is avoided (i.e., keeping plateau pressure < 30 cmH2O and driving pressure < 15 cmH2O); and a respiratory rate kept in a range between 8 and 16 breaths/min. In the comatose patient, the post-resuscitation goal remains, however, avoiding both hyperoxia and hypoxia and maintaining both normoxia, i.e., PaO2 10–13 kPa, and normocapnia, i.e., PaCO_2_ 4.5–6 pKa [14,17,18,49,50,64]. Nevertheless, in these patients, maintaining normocapnia with protective ventilation can be challenging due to the risk of using high PEEP together with increased dead space ventilation; often, hypercapnic acidosis occurs and can be particularly detrimental in the instance of cerebral injury, although the recent TAME trial has shown that targeting mild hypercapnia after cardiac arrest does not account for adverse neurological outcomes [65,66]. This highlights the need for a nuanced approach to ventilation in post-resuscitation care, tailored to the individual patient’s physiological needs [17,18,49].

## 6. Conclusions

This review underscores the importance of recognizing and managing ALI in patients undergoing CPR. It highlights the need for ongoing research to develop optimized CPR techniques and ventilation strategies, i.e., the use of a low level of PEEP or the new CPV, in order to achieve the following: to guarantee better gas exchanges while preventing airway closure; to reduce atelectasis in the dependent lung regions while reducing alveolar edema; to improve ventilation homogeneity and optimize the ventilation/perfusion ratio; and ultimately to minimize the risk of ALI. The prevalence of lung injury after cardiac arrest is high. Prolonged and forceful chest compressions, i.e., those performed through mechanical devices, may contribute to the development of lung edema and thus to a reduction in aerated lung volume and CRALE. CRALE and ALI are additional factors in the complex PCAS that must be considered in the management of resuscitated patients. Thus, it is reasonable to assess respiratory mechanics to aid the early identification of patients developing CRALE, characterized by lower respiratory system compliance and EELV and a higher dead space, since these patients may benefit from lung protective ventilation, i.e., a low tidal volume, the application of PEEP, plateau pressure < 30 cmH2O, and a respiratory rate of 8–16 breath/min, balanced with the potential increased risk of secondary brain injury induced by hypercapnia or high PEEP levels.

## Data Availability

Not applicable.

## Glossary

AD-mCC	mechanical chest compression with active decompression
ALI	acute lung injury
ARDS	acute respiratory distress syndrome
CC	chest compression
CPR	cardiopulmonary resuscitation
CPV	cardiopulmonary ventilation
CRALE	cardiopulmonary resuscitation-induced lung edema
Crs	respiratory system compliance
CT	computed tomography
EELV	end-expiratory lung volume
ICU	intensive care unit
IHCA	in-hospital cardiac arrest
HU	Hounsfield unit
mCC	mechanical chest compression
OR	odds ratio
PaO2/FiO2	oxygen arterial partial pressure/oxygen inspiratory fraction
PCAS	post-cardiac arrest syndrome
PEEP	positive end-expiratory pressure
OHCA	out-of-hospital cardiac arrest
Rrs	respiratory system resistance
ROSC	return of spontaneous circulation
VD/Vt	dead space
VILI	ventilator-induced lung injury
